# Joint estimation of survival and breeding probability in female dolphins and calves with uncertainty in state assignment

**DOI:** 10.1002/ece3.5693

**Published:** 2019-10-02

**Authors:** Pauline Couet, François Gally, Coline Canonne, Aurélien Besnard

**Affiliations:** ^1^ CNRS UM SupAgro IRD INRA UMR 5175 CEFE EPHE PSL Research University Montpellier France; ^2^ Groupe d'Etude des Cétacés du Cotentin Cherbourg‐Octeville France; ^3^ Direction Recherche et Expertise ONCFS Saint‐Benoit Auffargis France

**Keywords:** bottlenose dolphin, English Channel, multievent capture–recapture models, reproduction, survival, *Tursiops truncatus*

## Abstract

While the population growth rate in long‐lived species is highly sensitive to adult survival, reproduction can also significantly drive population dynamics. Reproductive parameters can be challenging to estimate as breeders and nonbreeders may vary in resighting probability and reproductive status may be difficult to assess. We extended capture–recapture (CR) models previously fitted for data on other long‐lived marine mammals to estimate demographic parameters while accounting for detection heterogeneity between individuals and state uncertainty regarding reproductive status. We applied this model to data on 106 adult female bottlenose dolphins observed over 13 years. The detection probability differed depending on breeding status. Concerning state uncertainty, offspring were not always sighted with their mother, and older calves were easier to detect than young‐of‐the‐year (YOY), respectively, 0.79 (95% CI 0.59–0.90) and 0.58 (95% CI 0.46–0.68). This possibly led to inaccurate reproductive status assignment of females. Adult female survival probability was high (0.97 CI 95% 0.96–0.98) and did not differ according to breeding status. Young‐of‐the‐year and 1‐year‐old calves had a significantly higher survival rate than 2‐year‐old (respectively, 0.66 CI 95% 0.50–0.78 and 0.45 CI 95% 0.29–0.61). This reduced survival is probably related to weaning, a period during which young are exposed to more risks since they lose protection and feeding from the mother. The probability of having a new YOY was high for breeding females that had raised a calf to the age of 3 or lost a 2‐year‐old calf (0.71, CI 95% 0.45–0.88). Yet, this probability was much lower for nonbreeding females and breeding females that had lost a YOY or a 1‐year‐old calf (0.33, 95% CI 0.26–0.42). The multievent CR framework we used is highly flexible and could be easily modified for other study questions or taxa (marine or terrestrial) aimed at modeling reproductive parameters.

## Graphical abstract

We develop an original parameterization of multievent capture–recapture model to deal with uncertainty in breeding state assignment and unknown age of the young to jointly estimate adult female survival, age‐dependant calf survival, state‐dependent breeding probability, and state‐dependent detection probabilities of female and calves. We took advantage of a 13 years (2004–2016) photo‐identification dataset collected on bottlenose dolphin (*Tursiops truncatus*) population to assess the relevance of the modeling approach.

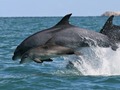

## 1 INTRODUCTION

Population dynamics studies rely on the estimation of several demographic parameters, such as survival by age class and fecundity (Caswell, 2001), which may contribute differently to the population growth rate (Kroon, Plaisier, Groenendael, & Caswell, 1986). In long‐lived species, several studies have demonstrated that the population growth rate, and thus population viability, is much more sensitive to variations in adult survival than to reproductive parameters (i.e., a modification of adult survival has much larger impact on population growth rate than a modification of reproductive parameters, see e.g., Oli & Dobson, 2003). Nonetheless, recent studies have pointed out that reproductive parameters should not be neglected when investigating the population dynamics of long‐lived species, especially in the context of population management (Manlik, Lacy, & Sherwin, 2018). The concept of environmental canalization (Gaillard & Yoccoz, 2003) states that demographic parameter with high sensitivity to population viability is less sensitive to environmental variations as this species usually developed some morphology, physiological, or behavioral adaptations to limit survival variations. Thus, in long‐lived species, the temporal variability of adult survival is quite low compared to juvenile survival or adult reproduction. In the absence of strong environmental perturbations (reduction of food availability, disease, destruction of core habitat) affecting the adult survival, variations in population trajectories can then be governed by juvenile survival or adult reproduction that can suffer higher level of variations (Genovart, Oro, & Tenan, 2018). Several studies have demonstrated that variations in reproductive parameters actually drive the population dynamics of long‐lived species (e.g., Beston, 2011 on bears; Gaillard, Festa‐Bianchet, & Yoccoz, 1998 on ungulates; Manlik et al., 2016 and Currey et al., 2009 on marine mammals; and Genovart et al., 2018 on long‐lived birds).

Studying reproductive parameters in wild populations of long‐lived species can, however, be challenging. One difficulty is that estimating these parameters relies on long‐term studies, ideally using individual longitudinal data. Another is that several traits are involved in reproduction, such as the proportion of breeding females or the reproductive success, and some of these are more difficult to estimate than others. Furthermore, the detection probability of breeders and nonbreeders could be different. For instance, the proportion of nonbreeding individuals is often extremely challenging to estimate as these animals may be difficult to detect (Bailey, Kendall, Church, & Wilbur, 2004), may occupy a different area than reproducing individuals, may spend little time on monitored sites, or may disperse outside of the study area. Reproductive success may also be challenging to estimate in some taxa, such as birds, as individuals that suffer early breeding failure may be difficult to observe (Ponchon, Iliszko, Grémillet, Tveraa, & Boulinier, 2017). Young individuals can also be difficult to detect and correctly assigned to the mother (Cheney, Thompson, & Cordes, 2019).

In this study, we were particularly interested in estimating the reproductive parameters of a population of bottlenose dolphins (*Tursiops truncatus*) with a new approach which could be used for other cetacean species. In wild cetacean populations, methods aiming at providing estimates of the reproductive rate, reproductive success, calf survival, and proportion of breeding female usually relied on raw count of breeding and nonbreeding adults, newborns, and juveniles, for purely descriptive statistics (see, for instance, Herzing, 1997; Rossi et al., 2017). Other studies used inferential statistical methods, like linear regression (Baker, O'Brien, McHugh, & Berrow, 2018) or general linear mixed models (Brough, Henderson, Guerra, & Dawson, 2016). These methods provide unbiased estimates only when the detection probability of animals is 1, or at least is not heterogeneous between individuals, years or sites. In studies of wild populations, this condition is rarely fulfilled, as individuals are seldom all sighted due to field constraints or animal behavior (Richman et al., 2014). This imperfect detection can occur on several levels. In case of reproductive status assessment, adult females usually have a detection probability of <1, except if the field effort is huge or the population very small. Equally, detection probability can also vary between reproductive states. For example, nonbreeding humpback whales most likely occupy deep waters offshore (Smultea, 1994). In such situations, the reproductive rate would be overestimated if breeding females are easier to detect, and underestimated if nonbreeding females are easier to detect. The same biases arise for estimating the proportion of breeding females. The second level of imperfect detection is related to juveniles. In cetaceans, observation conditions can be difficult: while an adult female may be seen, its true breeding status can be difficult to observe with certainty. Young can be hidden by the female or missed because they are small. Such omissions can lead to an underestimation of breeding rates if a calf dies before it can be observed, or an overestimation of calf mortality rates if an offspring is alive but not sighted.

Detection issues in estimating demographic parameters are traditionally dealt with capture–recapture models (CR hereafter, Lebreton, Burnham, Clobert, & Anderson, 1992). These methods are largely used to provide unbiased estimates of population size or survival probability (Kendall & Pollock, 1992), with a recent and growing focus on reproductive parameters for species breeding and calving on land (Desprez, Gimenez, McMahon, Hindell, & Harcourt, 2018; Desprez et al., 2014; Garnier, Gaillard, Gauthier, & Besnard, 2016; Oosthuizen, Pradel, Bester, & Bruyn, 2019). Cetacean populations are however more difficult to study than species breeding on land, as calving is at open sea, which poses specific challenges to estimate the reproductive parameters. Thus, the use of CR methods to study reproduction for those species is much less widespread (but see Cheney et al., 2019 and Rankin, Maldini, & Kaufman, 2014). The main constraint is the need for large datasets collected over several years on multiple animals that have been individually identified. Rankin et al. (2014) estimated calving intervals and breeding rate, as well as subadult and adult survival probability, but they do not account for uncertainty regarding breeding state assignment. Yet while a female observed with a calf is undoubtedly breeding, a female seen alone could be either nonbreeding or breeding but its calf was undetected. Only a few studies accounted for reproductive status uncertainty for marine mammal species with underwater calving (Cheney et al., 2019 on bottlenose dolphins and Kendall, Hines, & Nichols, 2003 on manatees). In this study, we aimed to estimate female survival probability, age‐dependent calf survival probability, and breeding probability, while accounting for detection probability and uncertainty in reproductive state assignment in cetacean populations. We also examined the impact of calf survival on the next breeding probability of a female, while considering age‐related calf detection probability. To our knowledge, no previous publicly available study has accounted for calf age and fate in breeding probability in cetacean species; indeed, in small cetacean species, maternal care can last several years after a calf's birth (Oftedal, 1997).

To this end, we studied a bottlenose dolphin (*Tursiops truncatus*) population in France. Of cetacean species, the bottlenose dolphin is one of the most studied. While much information is available concerning its distribution and abundance (Currey, Rowe, Dawson, & Slooten, 2008; Laporta, Fruet, & Secchi, 2016), reproductive parameters are more difficult to study, as it needs more field efforts and more detailed information on the reproductive state of the individuals. The vast majority of methods used to estimate reproductive parameters in this species did not account for detection probability (Baker et al., 2018; Brough et al., 2016; Haase & Schneider, 2001; Tezanos‐Pinto, Constantine, Mourão, Berghan, & Scott Baker, 2014) and thus are subject to the detection issues we detailed above. To address this, we used multievent CR models (Pradel, 2005) to estimate the probability of adult female survival and calf survival (age‐dependent), breeding probability (dependent on calf survival), female detection probability, and calf observation probability (given the state of the female, to account for uncertainty in breeding state assignment). For comparison, we also used a descriptive method based on Tezanos‐Pinto et al. (2014) to estimate calf survival probability while not taking detection issues into account.

## 2 METHODS

### 2.1 Sampling method and data collection

**Figure 1:**
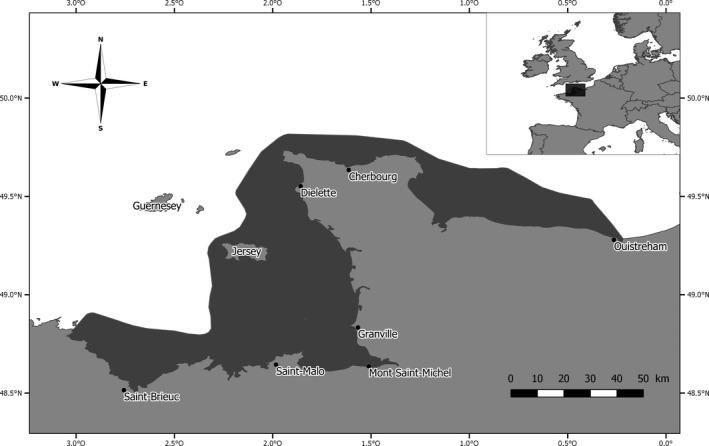
Map of the study area (dark gray) around the Normano‐Breton Gulf off the northern coast of France, where surveys were carried out on the bottlenose dolphin population between 2004 and 2016. The 40‐m bathymetric line defined the outer limit

We studied a population of bottlenose dolphins inhabiting the French waters of the Normano‐Breton Gulf in the English Channel (Figure 1). This population is resident year‐round, and the individuals are located close to the coast (Gally, 2014). The population size was estimated to be 389 individuals in 2016 (CI 95%: 367–412, Gally, Couet, & Riedmatten, 2018).

Each year from 2004 to 2016, we conducted dedicated boat surveys to survey the population. Some tests of systematic surveys were carried out in 2013 (unpublished results) but failed due to the large size of the area (approximately 7,000 km^2^, Gally, 2014), the unpredictable weather conditions for observations and the constrained induced by port of departure. These conditions made difficult to plan for a systematic sampling protocol. Thus, we opted for opportunistic surveys. Nonetheless, we avoided going over the same way during consecutive boat trips, and we prioritized the less sampled zones when the weather was nice, so that the zone was almost entirely covered each year. When a group of bottlenose dolphins was sighting, the boat approached the group slowly to allow photographs to be taken for identification. We tried to photograph all the animals in the group, and if young dolphins were present, efforts were made to have the young and the associated adult on the same picture. In this case, association means physical proximity, without space for another individual between the two animals. Sighting ended when all individuals were photographed or when the animals showed signs of boat avoidance or disturbance (e.g., extended diving, distancing, or other changes in behavior).

Data was collected all year‐round, but we constrained the analysis to the period between July and November, which corresponds both to the birth peak (summer/autumn season, Mann, Connor, Barre, & Heithaus, 2000) and the largest amount of data due to better weather conditions.

### 2.2 Photo identification

Using the photos, the individuals were identified based on scratches and notches on the dorsal fin, with some help from marks on the back and flanks. As young individuals usually do not have permanent mark, we only identified the adult female. An individual's sex was deducted by repeated association with a calf or by biopsy (previously done by Louis, 2014, using the SRY plus ZFX/ZFY fragments amplification method described in Rosel, 2003). For each resighting (identification in a photo) of a female, we document the presence or absence of a young individual, without identification of the young. They are easily distinguished from older animals by the size difference (Cheney, Wells, Barton, & Thompson, 2017), presence of fetal folds and lines, and infant position (Rossi et al., 2017). Thus, an identified female was classified as one of the following at each resighting: “female alone,” “female with a young‐of‐the‐year (YOY)” (age 0–1) or “female with a calf” (over age 1). It was not possible to estimate the age of a young after age 1.

When a female was resighted several times between July and November of the same year, we grouped these information to keep the most relevant information on breeding status (“event” sensu multivent CR method, see below) and to construct the CR histories. Hence, a female resighted at least once with a YOY was classified that year as a “female observed with a YOY.” A female was classified as a “female observed with a calf” when resighted at least twice with a calf, to limit the misclassification of a female mistakenly photographed next to a calf that was not hers. Since mother–calf association decreases with calf's age (Mann et al., 2000), an old calf could sometimes be seen close to another adult. In all other situations, the female was classified as a “female alone.”

### 2.3 Multievent capture–recapture models

The multievent CR framework (Pradel, 2005) distinguishes the visible layer (events that correspond to field observations) from the hidden layer (the true states). These observations can involve uncertainty regarding the latent state, modeled through the observation process. For instance, a breeding female could be misclassified as “alone” if her YOY or calf was missed (e.g., if it was underwater when its mother surfaced, if there were too many individuals to see it, etc.).

In the model, we defined six possible states to characterize a female: her breeding status (“NB” for nonbreeder or “B” for breeder), the age of the offspring if she was breeding (four age classes: “YOY” for a young‐of‐the‐year, “c1” for a 1‐year‐old calf, “c2” for a 2‐year‐old calf or “c3” for a 3‐year‐old calf, see Table 1), and a last possible state “D” if the female was dead (Table 1). The “nonbreeder” status meant that a female was not breeding a given year. The breeding status can change from year to year (see model description below). We assumed that a calf stayed with its mother until its third year (Mann et al., 2000), and that a given female could not have several young at the same time. This is corroborated by studies on calving intervals, which are estimated to be around 3–4 years in the bottlenose dolphin (Fruet, Genoves, Möller, Botta, & Secchi, 2015; Mann et al., 2000; Rossi et al., 2017; Steiner & Bossley, 2008). We defined four events that were coded in the capture histories: “0” if a female was not resighted; “1” if a female was sighted alone; “2” if a female was with a YOY, and “3” if a female was with a calf (over age 1).

**Table 1:** The six states and the four intermediate states of the multievent model for reproductive status

State description	
NB	Nonbreeding adult female
Byoy	Breeding adult female with a young‐of‐the‐year
Bc1	Breeding adult female with a 1‐year‐old calf
Bc2	Breeding adult female with a 2‐year‐old calf
Bc3	Breeding adult female with a 3‐year‐old calf
D	Dead female
**Intermediate state description**	
Byoy‐D	Breeding adult female that had lost her young‐of‐the‐year
Bc1‐D	Breeding adult female that had lost her 1‐year‐old calf
Bc2‐D	Breeding adult female that had lost her 2‐year‐old calf
Bc3‐leave	Breeding adult female that raised her calf to the age of 3

**Figure 2:**
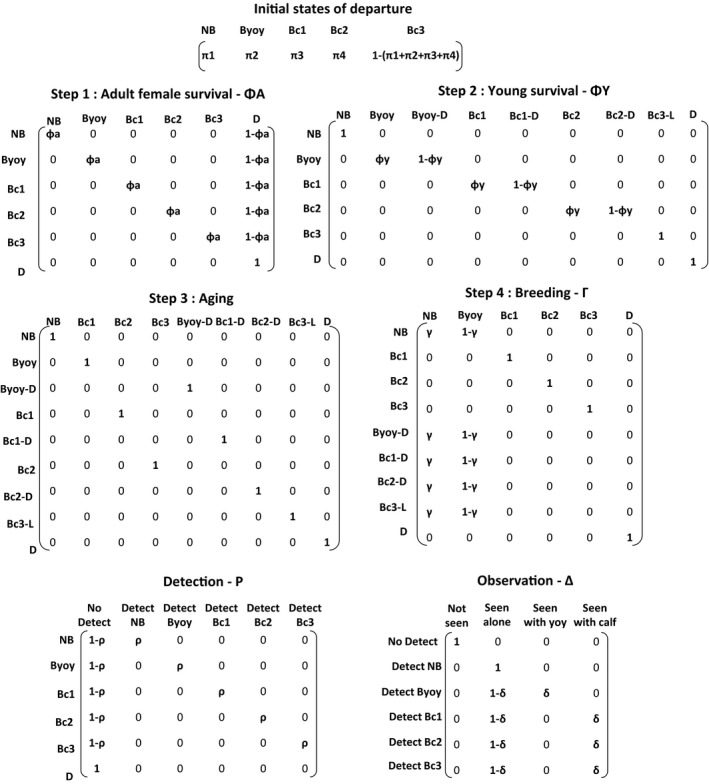
Modeling reproduction parameters for bottlenose dolphins: elementary matrices of state–state transitions and events (the states are described in Table 1). From the initial state of departure, the individual's state was successively updated through four modeling steps: (1) adult female survival, (2) young survival, (3) young aging, and (4) breeding. The rows correspond to the departure state at time *t* − 1 and the columns to the state of arrival at time *t*. The observation process was modeled with two steps: (1) detection and (2) observation

At their first sighting, females could be in one of the five states (Figure 2: initial states of departure), excluding the “Dead” state. They were entered in the dataset when they acquired distinctive natural markings and not according to a specific breeding state. The transition from one state to another from year *t* to *t* + 1 was split into four steps, which were converted into transition matrices: (1) female survival, (2) young survival, (3) young aging, and (4) breeding (Figure 2). Each step was conditional on all previous steps.

The first matrix was 6×6 and included female survival probability from *t* to *t* + 1. An adult female could survive with a probability of *φa* or die with a probability of 1 − *φa*. This survival probability could depend on a female's state at *t* (Figure 2, step 1: adult female survival).

The second matrix was 6×9 and modeled the YOY and calf survival probability (Figure 2, step 2: young survival). It included the probability of offspring to survive *φy* or die 1 − *φy*. When her offspring survived, the breeding female stayed in the same state, but when it died, she transitioned to an intermediate state (BYOY‐D, Bc1‐D or Bc2‐D: “D” here is dead offspring, Table 1). These intermediate states allowed us to keep the information about the death of young for the next matrices and to condition breeding probability to these states. For the 3‐year‐old calves, the survival probability could well be biased, as we could not discriminate between true mortality and calf emancipation from its mother (leading to a low association rate). To address this, we included an intermediate state, Bc3‐leave (Table 1), and fixed survival probability to 1 as this parameter was unidentifiable.

The third matrix was 9×9 and allowed transitions in age class for young individuals (Figure 2, step 3: aging). No parameters were estimated in this matrix as changes in age class for young were forced to 1 between *t* and *t* + 1.

The fourth matrix was 9×6 and modeled breeding probability (Figure 2, step 4: breeding). It included the probability of giving birth *γ* or not (1 − *γ*) and could depend on the state of the female. Notably it could depend on whether a female was a nonbreeder at *t* or had lost her offspring between *t* and *t* + 1 (see the “young survival” matrix).

Then the observation process, which linked events to states at time *t*, was split into two steps. The first step was a 6×6 matrix and modeled the detection probability ρ given the state of the female (Figure 2: detection). The second step was a 6×4 matrix and modeled the probability of observing a young individual *δ* or not 1 − *δ* for a breeding female (Figure 2: observation). We could not discriminate the age of a calf from the photos, so there were just two possible events for a breeding female with a calf between the age of 1 and 3: the calf was seen or not. However, the observation probability may differ between calf ages.

We started with a model in which all states have different values in initial probabilities (Π, Figure 2), adult and young survival (ΦA and ΦY, Figure 2), breeding probabilities (Γ, Figure 2), adult and young detection probabilities (*p* and Δ, Figure 2). We included a temporal variation in an additive way for the adult detection probability (*p*), as sampling effort was not equal between years. We then adopted a sequential backward selection procedure from this general model that we progressively simplified. We began by first simplifying the observation process, then detection, adult female survival, young survival and, finally, breeding probability. The hypotheses tested for each parameter are presented in Table 2. They resulted in 19 models (Appendix S1), which were ranked using Akaike information criteria adjusted for a small sample size (AICc, Burnham, & Anderson, 2002). We performed multistate goodness‐of‐fit tests (GOF) using UCARE V2.3.2 software (Choquet, Lebreton, Gimenez, Reboulet, & Pradel, 2009), and then fit the models in the E‐SURGE V1.9.0 program (Choquet, Rouan, & Pradel, 2009). A description of their implementation in E‐SURGE is available in Appendix S2.

**Table 2:** Detailed description of the assumptions tested for each parameter of the model. The meaning of abbreviations used is given in Table 1

Parameter	Hypothesis	Abbreviations
Observation process (Δ)	Differed between a breeding female with YOY versus a breeding female with a calf	Byoy versus Bc1, Bc2, and Bc3
	Differed between a breeding female with a YOY or a 1‐year‐old calf versus a breeding female with an older calf	Byoy and Bc1 versus Bc2 and Bc3
	Equal for every breeding female	
Detection probability (*p*) (always with temporal variation as an additive effect)	Differed between a nonbreeding female versus a female with a YOY versus a female with a calf	NB versus Byoy versus Bc1, Bc2, and Bc3
	Differed between a nonbreeding female versus a female with a YOY or a 1‐year‐old calf versus a female with a 2‐ or 3‐year‐old calf	NB versus Byoy and Bc1 versus Bc2 and Bc3
	Differed between a nonbreeding female or a female with a 2‐ or 3‐year‐old calf versus a female with a YOY or a 1‐year‐old calf	NB, Bc2, and Bc3 versus Byoy and Bc1
	Differed between a nonbreeding female versus a female with young	NB versus Byoy, Bc1 Bc2, and Bc3
	Equal for all state	
Adult survival (ΦA)	Differed between a nonbreeding female versus a breeding female	NB versus Byoy, Bc1 Bc2, and Bc3
	Equal for all state	
Young survival probability (ΦY)	Differed between YOY versus calf	Byoy versus Bc1 and Bc2
	Differed between YOY or 1‐year‐old calf versus a 2‐year‐old calf	Byoy and Bc1 versus Bc2
	Equal for all state	
Breeding probability (Γ)	Differed between a nonbreeding female versus a female that lost a YOY or a calf versus a female that raised a calf to the age of 3	NB versus Byoy‐D, Bc1‐D, and Bc2‐D versus Bc3‐leave
	Differed between a nonbreeding female versus a female that lost a YOY or a 1‐year‐old calf versus a female that lost a 2‐year‐old calf or raised a calf to the age of 3	NB versus Byoy‐D and Bc1‐D versus Bc2‐D and Bc3‐leave
	Differed between a nonbreeding female and a female that lost a 2‐year‐old calf or raised a calf to the age of 3 versus a female that lost a YOY or a 1‐year‐old calf	NB, Bc2‐D, and Bc3‐leave versus Byoy‐D and Bc1‐D
	Differed between a nonbreeding female versus a female that had a young	NB versus Byoy‐D, Bc1‐D, Bc2‐D, and Bc3‐leave
	Equal for all state	

### 2.4 Estimation of parameters not dealing with detection issues

We estimated some reproductive parameters (e.g., young survival probability) with descriptive methods in order to compare them to the estimations provided by the CR multievent methods. The approach used in this study was derived from those described in Tezanos‐Pinto et al. (2014). First‐year calf survival probability was calculated as the number of observed YOY that survived their first year divided by the total number of YOY observed over the full study period. The same logic was applied for the second‐year survival probability.

Like Tezanos‐Pinto et al. (2014), we were unable to discriminate the age of the young more precisely than YOY or calf (age 1–3). For this reason, the dataset was filtered so it only retained the females sighted with young individuals since the YOY state, in order to determine the calf's age in subsequent resightings. When a female previously sighted with a calf was resighted alone, Tezanos‐Pinto et al. (2014) assumed that the calf was dead as it did not reach weaning age. When a female was not resighted, the fate of the calf was unknown, so we did not consider this calf in the estimation of young survival probability using the descriptive approach.

## 3 RESULTS

From 2004 to 2016, we carried out 379 surveys and made 486 sightings (see Appendix S3 for further details on the number of events and photos for each year). A total of 106 adult females were identified (96 by association with young and 10 by biopsy) and sighted on 13,347 identification photos taken during this period. Only 13 of these females were never sighted with offspring between July and November; their sex was confirmed by biopsy or when sighted with young between December and June.

The results of the GOF test showed no evidence of lack of fit (*χ* ^2^ = 88.945, *p*‐value = .995). In the initial model selection process, two parameterizations of the observation probability were very close in terms of AICc scores. We thus kept both and conducted two parallel selection processes, each based on one of the two combinations (all models, ranked by AICc, are shown in Appendix S1). The best model included the effect of offspring age on observation probability and survival probability, the effects of breeding status and additive temporal variation on the detection process, the effect of the fate of the previous calf on breeding probability, and no effect on adult survival probability.

**Figure 3:**
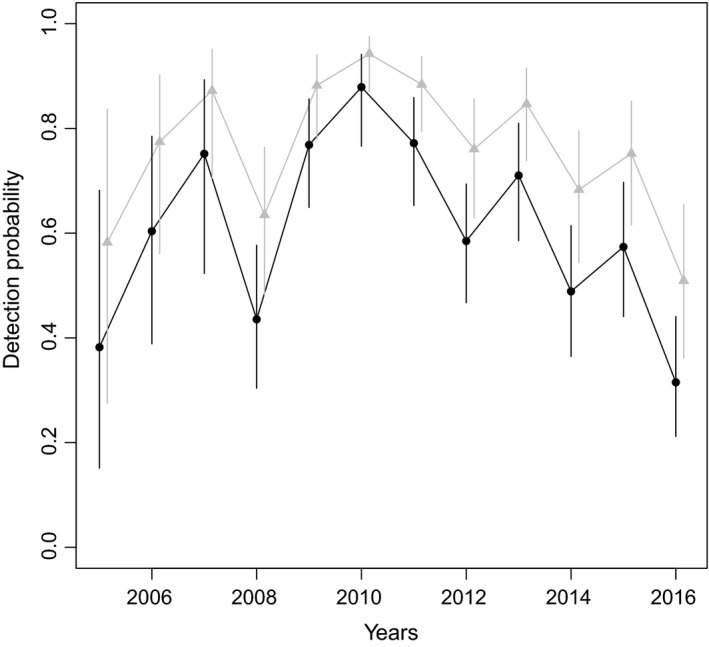
The detection probability of the adult female bottlenose dolphins in the study site (around the Normano‐Breton Gulf in the English Channel) between 2005 and 2016. The black line shows the estimates for nonbreeding females and breeding females with a 2‐ or 3‐year‐old calf. The gray line shows the estimates for breeding females with a YOY or a 1‐year‐old calf. Bars represent the 95% confidence intervals

The probability of missing an offspring when a female is actually breeding was high and was greater for YOY than for calves. The probability of observing a YOY was estimated at 0.58 (95% CI 0.46–0.68) and of observing a calf at 0.79 (95% CI 0.59–0.90). The detection probabilities of offspring were clearly below 1 and varied strongly over time (between 0.32 and 0.94). The detection probability also differed depending on the state of the female (Figure 3): females with a YOY or a 1‐year‐old calf had a greater detection probability than nonbreeding females or those with an older calf.

Female survival probability was estimated at 0.97 (95% CI 0.96–0.98) and did not differ between states. Young‐of‐the‐year and 1‐year‐old calf survival was estimated at 0.66 (95% CI 0.51–0.79), while 2‐year‐old calf survival was estimated at 0.45 (95% CI 0.30–0.62).

Breeding probability varied depending on the breeding state: nonbreeding females and females that had just lost their YOY or 1‐year‐old calf had a low probability of giving birth (0.33, 95% CI 0.26–0.42), whereas females that had lost their 2‐year‐old calf or raised it until the age of 3 had a greater probability of giving birth the next year (0.71, 95% CI 0.45–0.88).

Offspring survival probability estimated with the purely descriptive method was 0.53 (95% CI 0.42–0.63) for a YOY and 0.56 (95% CI 0.40–0.70) for a 1‐year‐old calf.

## 4 DISCUSSION

### 4.1 Uncertainty in the observation process

The estimated detection probability was <1 for all states, with great variation between years certainly related to the heterogeneity in field effort among years. Indeed, the highest detection probabilities corresponded to the years with the greatest number of surveys (43 in 2010 and 39 in 2011), and the lowest to the years with few surveys (21 in 2005, 27 in 2008 and 21 in 2016). Furthermore, detection probabilities widely varied depending on state (from 0.32 to 0.94). A breeding female with a YOY or a 1‐year‐old calf had the highest detection probability (0.68 95% CI 0.54–0.80 in 2014), as compared to a nonbreeding female or breeding female with a 2‐ or 3‐year‐old calf (0.49 95% CI 0.36–0.61 in 2014). This result may reflect observer bias, as there may have been a tendency to focus on females with a young calf in order to get accurate information on breeding status, resulting in taking less pictures of nonbreeding females or females with an older calf. In terms of the observation probability of offspring (i.e., of seeing the young of a sighted breeding female), this was low for YOY (0.58, 95% CI 0.46–0.68), and while substantially higher for calves (0.79, 95% CI 0.59–0.90), both indicate that offspring were not always sighted with their mother. The notable difference in observation probability in these two age classes is a little perplexing, as the detection probability for a female with a YOY was higher than for a female with a calf. It is possible that this could be explained by the fact that “sighted” in this study meant in reality “identified in pictures.” While observers made a greater effort to take pictures of females with a YOY (as reflected by the higher detection probability for these females), because of their small size (Rossi et al., 2017) and their proximity to their mother, which can mask them, YOY may remain unobserved in the pictures.

Imperfect detection of females and their young in turn affected the estimation of the other parameters. For example, the survival probability for a YOY or 1‐year‐old calf was estimated as 0.66 (95% CI 0.50–0.78) with the CR multievent framework. Yet the estimations using the descriptive method were much lower for the YOY (0.53 95% CI 0.42–0.63) and for the 1‐year‐old calf (0.56 95% CI 0.40–0.70). This discrepancy could be due to the fact that in the latter, when a female was seen alone, the calf was assumed dead, while it could have been alive and just not sighted. The CR approach revealed that 20% of the breeding females were sighted without their offspring, which indicates an underestimation of the offspring survival rate by the descriptive method.

These results illustrate the advantages of CR models in taking into account imperfect detection and unequal detection between states. In addition, employing a multievent framework in CR models allows the possibility of catching uncertainty in state assignation. Considering detection issues when estimating reproductive parameters has been recommended for terrestrial species (Beston, 2011; Gaillard et al., 1998), but it is necessary for cetacean as well (Cheney et al., 2019; Rankin et al., 2014).

### 4.2 Adult female survival

The survival probability of adult females did not differ between states and was estimated at 0.97 (CI 0.96–0.98). This high survival rate for adult female is expected for long‐lived mammals, for example in other populations of bottlenose dolphin: 0.96 in Scotland (95% CI 0.94–0.98, Arso Civil et al., 2019) and 0.94 in New Zealand (95% CI: 0.92–0.95, Currey et al., 2009). These rates are comparable to other CR studies on small cetaceans: orca (0.98, *SE* 0.01, Kuningas, Similä, & Hammond, 2013), and Indo‐Pacific bottlenose dolphin (0.93, 95% CI 0.88–0.96, Dulau, Estrade, & Fayan, 2017). We did not detect any effect of current reproduction status on survival probability. Trade‐offs between life‐history traits have been largely documented in theoretical studies (Stearns, 1989), with the theory stating that a limited supply of resources should lead to a balance between life‐history traits, as the energy invested in reproduction, for instance, would not be invested in maintenance or survival. Yet empirical studies have regularly demonstrated that such trade‐offs are hard to detect in long‐lived species (Moyes et al., 2011), since variation in individual quality could override the trade‐off in normal conditions (Richard, Toïgo, Appolinaire, Loison, & Garel, 2017) and be observed only when environmental conditions are harsh (Garnier et al., 2016). Having said that, evidence of a trade‐off between current and subsequent reproduction has been repeatedly reported in the literature on long‐lived species (Beauplet, Barbraud, Dabin, Küssener, & Guinet, 2006), and these questions deserve further study focusing on potential interindividual heterogeneity in cetaceans.

### 4.3 Offspring survival

The survival probability for a YOY or 1‐year‐old calf was estimated as 0.66 (CI 95% 0.50–0.78), and for a 2‐year‐old calf as 0.45 (CI 95% 0.29–0.61). Comparable survival probability for bottlenose dolphin YOYs has also been found in many other populations (some estimations are equivalent to ours, but most are higher, Table 3). Estimated survival probability between the age of 1 and 2 is rare, and is almost non‐existent for survival between the age of 2 and 3 (Table 3), although some studies have estimated the probability of raising a calf until its third year (instead of its survival probability after the age of 2, Table 3). Our estimation of 1‐year‐old calf survival was in the range of the available estimations (Table 3), but the estimation for 2‐year‐old calf was much lower than calf's survival in other populations (Table 3). Yet, survival probability estimates between the age of 1 and 2 are rare and are almost non‐existent for survival between the age of 2 and 3 (Table 3).

**Table 3:** Estimated apparent survival of YOY (young‐of‐the‐year) and calves (age 1 and 2) in other bottlenose dolphin populations from selected studies

	Species	Young apparent survival		
		YOY	1‐year‐old	2‐year‐old
Wells and Scott (1990)	*Tursiops truncatus*	0.80 (*SD* 0.07)	–	–
Mann et al. (2000)	*Tursiops* sp.	0.71	0.82	0.97
Haase and Schneider (2001)	*Tursiops truncatus*	0.8	–	–
Kogi et al. (2004)	*Tursiops aduncus*	0.87	–	–
Steiner & Bossley (2008)	*Tursiops aduncus*	0.70	0.54	–
Currey et al. (2009)	*Tursiops* sp.	0.86 (0.69–0.95)^a^ 0.38 (0.21–0.58)^a^		
Henderson et al. (2014)	*Tursiops truncatus*	0.67	To the age of 3:0.4	
Tezanos‐Pinto et al. (2014)	*Tursiops truncatus*	0.66 (0.52–0.79)^b^ 0.48 (0.34–0.63)^b^	0.85 (0.76–0.98)^b^ 0.41 (0.25–0.59)^b^	–
Fruet et al. (2015)	*Tursiops truncatus*	0.86 (0.75–0.92)	0.86 (0.75–0.92)	–
Robinson et al. (2017)	*Tursiops truncatus*	From age 0 to 2 or 3:0.83		
Rossi et al. (2017)	*Tursiops truncatus*	0.75	–	–
Arso Civil et al. (2019)	*Tursiops truncatus*	0.87 (0.79–0.92)	0.98 (0.78–0.99)	0.88 (0.71–0.96)
Cheney et al. (2019)	*Tursiops truncatus*	0.78 (0.53–0.92)^c^ 0.93 ( 0.82–0.98)^c^	0.32 (0.19–0.48)^c^ 0.55 (0.44–0.65)^c^	

a Currey et al. (2009), first period of the study, 1994–2001; and then the second period 2002–2008.

b Tezanos‐Pinto et al. (2014) supposed that all calves never resighted were alive (first line) and then supposed that all calves never resighted were dead (second line).

c Cheney et al. (2019), first period of the study in 2001; then the second period in 2016.

Higher survival of young calves than older ones have also been reported in other mammal species with late weaning (several years after birth), such as elephants (Mumby, Courtiol, Mar, & Lummaa, 2013; Young & Van Aarde, 2010) and great apes (Furuichi et al., 1998; Thompson, Kahlenberg, Gilby, & Wrangham, 2007). The weaning process is usually cited as a contributing factor for this declining survival. In the case of the bottlenose dolphin, weaning happens at around the age of 2 (Kastelein, Vaughan, Walton, & Wiepkema, 2002). This critical period involves leaving the mother's protection, stopping suckling to seek its own food, and socializing with other individuals (Lee, 1996). However, another process could have biased our estimation of YOY survival: very early offspring mortality. Of first‐year young that do not survive, most seem to die during their first months of life (Mumby et al., 2013 for elephants, Henderson, Dawson, Currey, Lusseau, & Schneider, 2014 for bottlenose dolphins). In this case, early mortality could have led to the overestimation of YOY survival probability and an underestimation of the proportion of breeding females, as we could have missed some newborns if they died before our surveys.

### 4.4 Breeding probability

The best CR model showed that breeding probability depended on the previous reproductive state. Breeding probability was high for breeding females that had raised a calf to the age of 3 or lost a 2‐year‐old calf (0.71, CI 95% 0.45–0.88). Lower breeding probability was found for nonbreeding females and breeding females that had lost a YOY or a 1‐year‐old calf (0.33, 95% CI 0.26–0.42). Similar results were found by Kendall, Langtimm, Beck, and Runge (2004) on manatees, in which the reproductive rate of females with a YOY was low (0.016, *SE* 0.015) and was higher for females with an older calf (0.38 *SE* 0.045). However, in contrast to our findings, they found that manatee females with no calf had a high reproductive rate, equal to a female with an older calf. Two factors may account for this discrepancy. First, in our study the nonbreeding females may have been younger and older individuals, whose fecundity is often lower (inexperience and senescence, Beauplet et al., 2006). Second, it seems that the females that had lost offspring needed a resting period before investing in a new calf. The lactation period requires more energy than gestation (Kastelein et al., 2002; Lee, 1996), hence the probability of a new birth is lower if an adult female loses an unweaned calf (as the female may need a resting period (Fedigan & Rose, 1995). While two consecutive births for a given female have been reported in some studies (Kogi, Hishii, Imamura, Iwatani, & Dudzinski, 2004; Steiner & Bossley, 2008), these were occasional and seemed to involve only a few breeding females. In our dataset, consecutive sightings of YOY for a given female happened only six times over about 190 breeding attempts.

Adult female bottlenose dolphins invest heavily over several years to raise their offspring (Arso Civil, Cheney, Quick, Thompson, & Hammond, 2017), like other mammal species such as elephants (Lee, 1987) and chimpanzees (Van Lawick‐Goodall, 1968). Providing such intensive parental care may have an impact on the female's subsequent reproduction (cost of reproduction, Paterson, Rotella, Link, & Garrott, 2018), as well as other demographic traits (trade‐offs) such as survival, which is the predominant trait for long‐lived species. In this study, we did not observe a trade‐off between current reproductive status and survival (see the “Adult survival” section). We also did not detect a cost of the current reproduction on the next one. We would have expected a higher breeding probability for nonbreeding females in case of a reproductive cost, but this was not the case here. The difference in breeding probabilities is likely to be explained by the age of the female or by heterogeneity in a female's quality in terms of breeding or offspring survival probabilities (Moyes et al., 2011; Richard et al., 2017). The models we used could easily be modified to incorporate heterogeneity and age effect if the amount of data was sufficient.

### 4.5 Perspectives

Despite its useful findings, our study suffers certain limitations that would deserve more attention in the future. First, the dataset consisted almost entirely of females that bred at least once. Indeed, as there is no sexual dimorphism in bottlenose dolphins, females were identified by their association with a YOY or a calf. Only 10 females with no association to a calf over the study period were included in our dataset. This could result in an overestimation of breeding probability, as females that never breed would not be accounted for in the analysis. Systematic genetic analysis might lower this potential bias. Second, we had no information on the age of the females, yet the literature on long‐lived species cites many examples of age‐related breeding and fecundity parameters (Moyes et al., 2011). Relying on natural markings for identification, which was necessary for resighting an animal over the years, may have led individuals of a certain age class or social status to be targeted, as the number of marks might be related to these parameters. Unfortunately, there is no easy solution to this problem, which is a limitation of all demographic analyses of cetacean populations. Some alternative to natural marks has recently emerged based on facial recognition of dolphins (Genov, Centrih, Wright, & Wu, 2018) and should be considered for identifying young animals in the future allowing for a more direct approach to estimate young survival probabilities.

## 5 CONCLUSION

Reproductive parameters are difficult to assess for long‐lived species. Our results confirmed that considering imperfect detection via CR models was essential to obtain unbiased demographic parameters. In particular, multievent CR models can allow the simultaneous estimation of breeding probability, offspring survival probability and adult survival probability while accounting for uncertainty in reproductive status assignment. However, this method requires individual identification and, for long‐lived species, multiple years of surveys. Such data are relatively rare in cetacean species.

The multievent CR framework we employed is highly flexible and could be easily modified to fit other situations and other taxa (marine or terrestrial) aiming at modeling reproductive parameters. For instance, individual heterogeneity in resighting probability (e.g., various behaviors to evade observation/camera trap) or in demographic traits can be easily incorporated using mixture models (Pledger & Phillpot, 2008). While such CR models are commonly used to study survival and abundance (Kendall & Pollock, 1992), they are much less widespread to study reproduction. It is increasingly used for terrestrial species (Garnier et al., 2016) and marine species that breed on land (Desprez et al., 2018, 2014; Garnier et al., 2016; Oosthuizen et al., 2019), but it is still rare for species breeding at sea (though see Cheney et al., 2019 and Rankin et al., 2014). We advocate for their systematic use in future studies on population dynamics to account for imperfect detection and state uncertainty assignment.

## CONFLICT OF INTEREST

None declared.

## AUTHOR CONTRIBUTIONS

PC, AB and CC conceived the ideas and designed methodology; FG collected the data; PC and CC analyzed the data; PC and AB led the writing of the manuscript. All authors contributed critically to the drafts and gave final approval for publication.

## Data Availability

The raw data are available in ObsenMer database (http://www.obsenmer.org/) and the CR matrix is available from the Dryad Digital Repository: https://doi.org/10.5061/dryad.5bh0587.
