# Supplementary material for: Joint estimation of survival and breeding probability in female dolphins and calves with uncertainty in state assignment
Source: Ecol Evol. 2019 Oct 2;9(23):13043–55. doi: 10.1002/ece3.5693 (PMC6912916; doi:10.1002/ece3.5693)
Supplement: Supplementary file 1 [file ECE3-9-13043-s001.docx]

**APPENDIX S1**: Models from the two selection procedures. In the initial model selection process, two parametrizations of the observation probability were very close in terms of AICc scores. We thus kept both and carried out two parallel selection processes, each with one of the two combinations.

Model abbreviations:

**ΦA**: adult female survival

**ΦY**: YOY and calf survival

**Γ**: breeding probability

**P**: detection probability

**Δ**: offspring observation probability

“.”: all equal

“allStates”: all different

NB: non-breeding female

Byoy: breeding female with a YOY

Bc1: breeding female with a 1-year-old calf

Bc1-D: breeding female that lost a 1-year-old calf

Bc2: breeding female with a 2-year-old calf

Bc2-D: breeding female that lost a 2-year-old calf

Bc3: breeding female with a 3-year-old calf

Bc3-L: breeding female that raises a calf to the age of 3

t: additive time effect on capture

Appendix S1: Table S1: Models from the first selection procedure, in which we kept **Δ** (.) for the observation hypothesis.

| Rank | Model | Np | AICc | ΔAICc |
| --- | --- | --- | --- | --- |
| 1 | **ΦA** (.),**ΦY** (Byoy Bc1 vs Bc2), **Γ** (NB Bc2-D Bc3-L vs Byoy-D Bc1-D), **P** (t, NB Bc2 Bc3 vs Byoy Bc1), **Δ** (.) | 23 | 2358.93 | 0.00 |
| 2 | **ΦA** (.),**ΦY** (Byoy Bc1 vs Bc2), **Γ** (allStates),**P** (t, NB Bc2 Bc3 vs Byoy-D Bc1), **Δ** (.) | 26 | 2360.35 | 1.42 |
| 3 | **ΦA** (.),**ΦY** (Byoy Bc1 vs Bc2), **Γ** (NB vs Byoy-D Bc1-D Bc2D Bc3-L),**P** (t, NB Bc2 Bc3 vs Byoy Bc1), **Δ** (.) | 23 | 2360.61 | 1.68 |
| 4 | **ΦA** (.),**ΦY** (Byoy Bc1 vs Bc2), **Γ** (NB vs Bc2-D Bc3-L vs Byoy-D Bc1-D), **P** (t, NB Bc2 Bc3 vs Byoy Bc1), **Δ** (.) | 24 | 2360.70 | 1.77 |
| 5 | **ΦA** (.),**ΦY** (Byoy Bc1 vs Bc2), **Γ** (NB vs Byoy-D Bc1-D Bc2-D vs Bc3-L),**P** (t, NB Bc2 Bc3 vs Byoy Bc1), **Δ** (.) | 24 | 2361.42 | 2.49 |
| 6 | **ΦA** (.),**ΦY** (Byoy vs Bc1 Bc2), **Γ** (allStates),**P** (t, NB Bc2 Bc3 vs Byoy Bc1), **Δ** (.) | 26 | 2361.54 | 2.61 |
| 7 | **ΦA** (.),**ΦY** (allStates), **Γ** (allStates),**P** (t, NB Bc2 Bc3 vs Byoy Bc1), **Δ** (.) | 27 | 2362.03 | 3.10 |
| 8 | **ΦA** (NB vs Byoy Bc1 Bc2 Bc3),**ΦY** (allStates), **Γ** (allStates),**P** (t, NB Bc2 Bc3 vs Byoy Bc1), **Δ** (.) | 28 | 2364.08 | 5.15 |
| 9 | **ΦA** (.),**ΦY** (.), **Γ** (allStates),**P** (t, NB Bc2 Bc3 vs Byoy Bc1), **Δ** (.) | 25 | 2364.11 | 5.18 |
| 10 | **ΦA** (.),**ΦY** (Byoy Bc1 vs Bc2), **Γ** (.),**P** (t, NB Bc2 Bc3 vs Byoy Bc1), **Δ** (.) | 22 | 2366.79 | 7.86 |
| 11 | **ΦA** (allStates),**ΦY** (allStates), **Γ** (allStates),**P** (t, NB Bc2 Bc3 vs Byoy Bc1), **Δ** (.) | 31 | 2368.17 | 9.24 |
| 12 | **ΦA** (allStates),**ΦY** (allStates), **Γ** (allStates),**P** (t, NB vs Byoy Bc1 vs Bc2 Bc3), **Δ** (.) | 32 | 2370.01 | 11.08 |
| 13 | **ΦA** (allStates),**ΦY** (allStates), **Γ** (allStates),**P** (t, allStates), **Δ** (.) | 34 | 2372.02 | 13.09 |
| 14 | **ΦA** (allStates),**ΦY** (allStates), **Γ** (allStates),**P** (t, NB vs Byoy Bc1 Bc2 Bc3), **Δ** (.) | 31 | 2372.36 | 13.43 |
| 15 | **ΦA** (allStates),**ΦY** (allStates), **Γ** (allStates),**P** (t, allStates), **Δ** (Byoy vs Bc1 Bc2 Bc3) | 35 | 2372.90 | 13.97 |
| 16 | **ΦA** (allStates),**ΦY** (allStates), **Γ** (allStates),**P** (t, NB vs Byoy vs Bc1 Bc2 Bc3), **Δ** (.) | 32 | 2374.00 | 15.07 |
| 17 | **ΦA** (allStates),**ΦY** (allStates), **Γ** (allStates),**P** (t, .), **Δ** (.) | 30 | 2374.21 | 15.28 |
| 18 | **ΦA** (allStates),**ΦY** (allStates), **Γ** (allStates),**P** (t, allStates), **Δ** (Byoy Bc1 vs Bc2 Bc3) | 35 | 2374.24 | 15.31 |
| 19 | **ΦA** (allStates),**ΦY** (allStates), **Γ** (allStates),**P** (t, allStates), **Δ** (allStates) | 37 | 2377.23 | 18.30 |

Appendix S1: Table S2: Models from the second selection procedure, in which we kept **Δ** (Byoy vs Bc1 Bc2 Bc3) for the observation hypothesis.

| Rank | Model | Np | AICc | ΔAICc |
| --- | --- | --- | --- | --- |
| 1 | **ΦA** (.),**ΦY** (Byoy Bc1 vs Bc2), **Γ** (NB Bc2-D Bc3-L vs Byoy-D Bc1-D), **P** (t, NB Bc2 Bc3 vs Byoy Bc1), **Δ** (Byoy vs Bc1 Bc2 Bc3) | 24 | 2356.81 | 0 |
| 2 | **ΦA** (.),**ΦY** (Byoy Bc1 vs Bc2), **Γ** (NB vs Bc2-D Bc3-L vs Byoy-D Bc1-D), **P** (t, NB Bc2 Bc3 vs Byoy Bc1), **Δ** (Byoy vs Bc1 Bc2 Bc3) | 25 | 2358.88 | 2.07 |
| 3 | **ΦA** (.),**ΦY** (Byoy Bc1 vs Bc2), **Γ** (allStates),**P** (t, NB Bc2 Bc3 vs Byoy Bc1), **Δ** (Byoy vs Bc1 Bc2 Bc3) | 27 | 2360.15 | 3.34 |
| 4 | **ΦA** (.),**ΦY** (.), **Γ** (allStates),**P** (t, NB Bc2 Bc3 vs Byoy Bc1), **Δ** (Byoy vs Bc1 Bc2 Bc3) | 26 | 2360.35 | 3.54 |
| 5 | **ΦA** (.),**ΦY** (Byoy vs Bc1 Bc2), **Γ** (allStates),**P** (t, NB Bc2 Bc3 vs Byoy Bc1), **Δ** (Byoy vs Bc1 Bc2 Bc3) | 27 | 2361.13 | 4.32 |
| 6 | **ΦA** (.),**ΦY** (allStates), **Γ** (allStates),**P** (t, NB Bc2 Bc3 vs Byoy Bc1), **Δ** (Byoy vs Bc1 Bc2 Bc3) | 28 | 2362.18 | 5.37 |
| 7 | **ΦA** (.),**ΦY** (Byoy Bc1 vs Bc2), **Γ** (NB vs Byoy-D Bc1-D Bc2D Bc3-L),**P** (t, NB Bc2 Bc3 vs Byoy Bc1), **Δ** (Byoy vs Bc1 Bc2 Bc3) | 24 | 2362.38 | 5.57 |
| 8 | **ΦA** (.),**ΦY** (Byoy Bc1 vs Bc2), **Γ** (NB vs Byoy-D Bc1-D Bc2-D vs Bc3-L),**P** (t, NB Bc2 Bc3 vs Byoy Bc1), **Δ** (Byoy vs Bc1 Bc2 Bc3) | 25 | 2363.57 | 6.76 |
| 9 | **ΦA** (NB vs Byoy Bc1 Bc2 Bc3),**ΦY** (allStates), **Γ** (allStates),**P** (t, NB Bc2 Bc3 vs Byoy Bc1), **Δ** (Byoy vs Bc1 Bc2 Bc3) | 29 | 2364.28 | 7.47 |
| 10 | **ΦA** (.),**ΦY** (Byoy Bc1 vs Bc2), **Γ** (.),**P** (t, NB Bc2 Bc3 vs Byoy Bc1), **Δ** (Byoy vs Bc1 Bc2 Bc3) | 23 | 2365.68 | 8.87 |
| 11 | **ΦA** (allStates),**ΦY** (allStates), **Γ** (allStates),**P** (t, NB Bc2 Bc3 vs Byoy Bc1), **Δ** (Byoy vs Bc1 Bc2 Bc3) | 32 | 2367.88 | 11.07 |
| 12 | **ΦA** (allStates),**ΦY** (allStates), **Γ** (allStates),**P** (t, NB vs Byoy Bc1 vs Bc2 Bc3), **Δ** (Byoy vs Bc1 Bc2 Bc3) | 33 | 2369.78 | 12.97 |
| 13 | **ΦA** (allStates),**ΦY** (allStates), **Γ** (allStates),**P** (t, NB vs Byoy Bc1 Bc2 Bc3), **Δ** (Byoy vs Bc1 Bc2 Bc3) | 32 | 2370.47 | 13.66 |
| 14 | **ΦA** (allStates),**ΦY** (allStates), **Γ** (allStates),**P** (t, .), **Δ** (Byoy vs Bc1 Bc2 Bc3) | 31 | 2371.66 | 14.85 |
| 15 | **ΦA** (allStates),**ΦY** (allStates), **Γ** (allStates),**P** (t, allStates), **Δ** (.) | 34 | 2372.02 | 15.21 |
| 16 | **ΦA** (allStates),**ΦY** (allStates), **Γ** (allStates),**P** (t, NB vs Byoy vs Bc1 Bc2 Bc3), **Δ** (Byoy vs Bc1 Bc2 Bc3) | 33 | 2372.56 | 15.75 |
| 17 | **ΦA** (allStates),**ΦY** (allStates), **Γ** (allStates),**P** (t, allStates), **Δ** (Byoy vs Bc1 Bc2 Bc3) | 35 | 2372.90 | 16.09 |
| 18 | **ΦA** (allStates),**ΦY** (allStates), **Γ** (allStates),**P** (t, allStates), **Δ** (Byoy Bc1 vs Bc2 Bc3) | 35 | 2374.24 | 17.43 |
| 19 | **ΦA** (allStates),**ΦY** (allStates), **Γ** (allStates),**P** (t, allStates), **Δ** (allStates) | 37 | 2377.23 | 20.42 |

**APPENDIX S2**: Implementation of the multievent CR models in the E-SURGE program (E-SURGE can be downloaded free from the CEFE website: <http://www.cefe.cnrs.fr/en/biostatistics-and-biology-of-populations/software>).

First, we defined the states and events and provided the structure of the transition matrices using the GEPAT interface. The GEPAT structure remains the same for all models. The hypotheses were tested with the GEMACO interface. The symbol ‘*’ indicates the complement of the sum of a row, while the symbol ‘-’ indicates inactive cells corresponding to a probability of 0. Note that the same letter in two cells does not imply equality in parameter values.

**Initial states of departure**


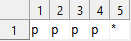


**Initial states of departure**

All states were considered, except the state ‘Dead’, which does not appear in GEPAT by default.

- 1: **NB** = non-breeding female
- 2: **Byoy** = breeding female with a young-of-the-year (YOY)
- 3: **Bc1** = breeding female with a 1-year-old calf (c1)
- 4: **Bc2** = breeding female with a 2-year-old calf (c2)
- 5: **Bc3** = breeding female with a 3-year-old calf (c3)

**Step 1: Adult female survival**

**
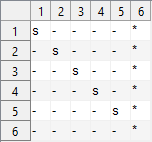
**

**Step 1: Adult female survival**

The six departure states (in rows) considered in this matrix were:

- Row 1: **NB** = non-breeding female alive at time *t-1*
- Row 2: **Byoy** = breeding female alive at time *t-1* with a YOY
- Row 3: **Bc1** = breeding female alive at time *t-1* with a 1-year-old calf
- Row 4: **Bc2** = breeding female alive at time *t-1* with a 2-year-old calf
- Row 5: **Bc3**= breeding female alive at time *t-1* with a 3-year-old calf
- Row 6: **D** = dead female at time *t-1*

The six arrival states (in columns) considered were:

- Column 1: **NB** = non-breeding female alive at time *t*
- Column 2: **Byoy** = breeding female alive at time *t* with a YOY
- Column 3: **Bc1** = breeding female alive at time *t* with a 1-year-old calf
- Column 4: **Bc2** = breeding female alive at time *t* with a 2-year-old calf
- Column 5: **Bc3** = breeding female alive at time *t* with a 3-year-old calf
- Column 6: **D** = dead female at time *t*

**Step 2: Offspring survival**

**
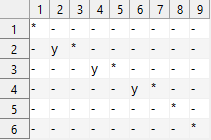
**

**Step 2: Offspring survival**

The six departure states (in rows) considered in this matrix were:

- Row 1: **NB** = non-breeding female at time *t-1*
- Row 2: **Byoy** = breeding female with a YOY alive at time *t-1*
- Row 3: **Bc1** = breeding female with a 1-year-old calf alive at time *t-1*
- Row 4: **Bc2** = breeding female with a 2-year-old calf alive at time *t-1*
- Row 5: **Bc3** = breeding female with a 3-year-old calf alive at time *t-1*
- Row 6: **D** = dead female at time *t-1*

The nine arrival states (in columns) considered were:

- Column 1: **NB** = non-breeding female at time *t*
- Column 2: **Byoy** = breeding female with a YOY alive at time *t*
- Column 3: **Byoy-D** = breeding female with a dead YOY at time *t*
- Column 4: **Bc1**= breeding female with a 1-year-old calf alive at time *t*
- Column 5: **Bc1-D** = breeding female with a dead 1-year-old calf at time *t*
- Column 6: **Bc2** = breeding female with a 2-year-old calf alive at time *t*
- Column 7: **Bc2-D** = breeding female with a dead 2-year-old calf at time *t*
- Column 8: **Bc3-L** = breeding female with an emancipated 3-year-old calf (i.e. had left the mother) at time *t*
- Column 9: **D** = dead female at time *t*

**Step 3: Ageing**


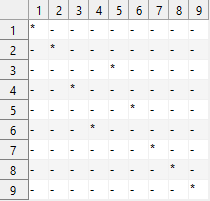


**Step 3: Ageing**

This matrix allowed offspring to transition in age class. No parameters were estimated in this matrix as changes in age class for calves was forced to 1 (*) between *t* and *t+1*.

The nine departure states (in rows) considered in this matrix were:

- Row 1: **NB** = non-breeding female at time *t-1*
- Row 2: **Byoy** = breeding female with a YOY alive at time *t-1*
- Row 3: **Byoy-D** = breeding female with a dead YOY at time *t-1*
- Row 4: **Bc1** = breeding female with a 1-year-old calf alive at time *t-1*
- Row 5: **Bc1-D** = breeding female with a dead 1-year-old calf at time *t-1*
- Row 6: **Bc2** = breeding female with a 2-year-old calf alive at time *t-1*
- Row 7: **Bc2-D** = breeding female with a dead 2-year-old calf at time *t-1*
- Row 8: **Bc3-L** = breeding female with an emancipated 3-year-old calf (i.e. had left the mother) at time *t-1*
- Row 9: **D** = dead female at time *t-1*

The nine arrival states (in columns) considered were:

- Column 1: **NB** = non-breeding female at time *t*
- Column 2: **Bc1** = breeding female with a 1-year-old calf alive at time *t*
- Column 3: **Bc2** = breeding female with a 2-year-old calf alive at time *t*
- Column 4: **Bc3** = breeding female with a 3-year-old calf alive at time *t*
- Column 5: **Byoy-D** = breeding female with a dead YOY at time *t*
- Column 6: **Bc1-D** = breeding female with a dead 1-year-old calf at time *t*
- Column 7: **Bc2-D** = breeding female with a dead 2-year-old calf at time *t*
- Column 8: **Bc3-L** = breeding female with an emancipated 3-year-old calf (i.e. had left the mother) at time *t*
- Column 9: **D** = dead female at time *t*

**Step 4: Breeding**

**
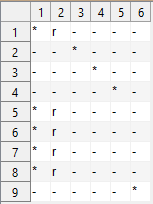
**

**Step 4: Breeding**

The nine departure states (in rows) considered in this matrix were:

- Row 1: **NB =** non-breeding female at time *t*
- Row 2: **Bc1** = breeding female with a 1-year-old calf alive at time *t*
- Row 3: **Bc2** = breeding female with a 2-year-old calf alive at time *t*
- Row 4: **Bc3** = breeding female with a 3-year-old calf alive at time *t*
- Row 5: **Byoy-D** = breeding female with a dead YOY at time *t*
- Row 6: **Bc1-D** = breeding female with a dead 1-year-old calf at time *t*
- Row 7: **Bc2-D** = breeding female with a dead 2-year-old calf at time *t*
- Row 8: **Bc3-L** = breeding female with an emancipated 3-year-old calf (i.e. had left the mother) at time *t*
- Row 9: **D** = dead female at time *t*

The six arrival states (in columns) considered were:

- Column 1: **NB** = non-breeding female at time *t*
- Column 2: **Byoy** = breeding female with a YOY at time *t*
- Column 3: **Bc1** = breeding female with a 1-year-old calf at time *t*
- Column 4: **Bc2** = breeding female with a 2-year-old calf at time *t*
- Column 5: **Bc3** = breeding female with a 3-year-old calf at time *t*
- Column 6: **D** = dead female at time *t*

**Detection**


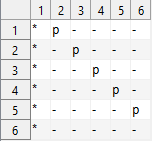


**Detection**

Detection probability given the state of the female.

The six departure states (in rows) considered in this matrix were:

- Row 1: **NB** = non-breeding female at time *t*
- Row 2: **Byoy** = breeding female at time *t* with a YOY
- Row 3: **Bc1** = breeding female at time *t* with a 1-year-old calf
- Row 4: **Bc2** = breeding female at time *t* with a 2-year-old calf
- Row 5: **Bc3** = breeding female at time *t* with a 3-year-old calf
- Row 6: **D** = dead female at time *t*

The six arrival states (in columns) considered were:

- Column 1: **Not detected**
- Column 2: **Detect NB** = non-breeding female detected at time *t*
- Column 3: **Detect Byoy** = breeding female with a YOY detected at time *t*
- Column 4: **Detect Bc =**, breeding female with a 1-year-old calf detected at time *t*
- Column 5: **Detect Bc2** = breeding female with a 2-year-old calf detected at time *t*
- Column 6: **Detect Bc3** = breeding female with a 3-year-old calf detected at time *t*

**Observation**

**
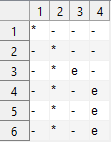
**

**Observation**

Probability of observing the offspring of detected breeding females.

The six departure states (in rows) considered in this matrix are:

- Row 1: **Not detected**
- Row 2: **Detect NB** = non-breeding female detected at time *t*
- Row 3: **Detect Byoy** = breeding female with a YOY detected at time *t*
- Row 4: **Detect Bc1** = breeding female with a 1-year-old calf detected at time *t*
- Row 5: **Detect Bc2** = breeding female with a 2-year-old calf detected at time *t*
- Row 6: **Detect Bc3** = breeding female with a 3-year-old calf detected at time *t*

The four possible events (in columns) were:

- Column 1: **0** = not sighted at time *t*
- Column 2: **1** = female sighted alone at time *t*
- Column 3: **2** = female sighted with a YOY at time *t*
- Column 4: **3** = female sighted with a calf (between the age of 1 and 3) at time *t*

Next, the GEMACO interface allowed effects of interest to be specified for each parameter (e.g. female survival, young survival, breeding, detection and observation). We tested the following hypotheses on the model.

**Step 1: Female survival**

1. all states different

**from**

1. survival probability depended on breeding state (non-breeding female versus breeding female)

**from(1,2:5)**

1. constant survival probability (no difference between states)

**i**

**Step 2: Young survival**

1. all states different

**from**

1. differs between YOY versus calf

**from(2,3 4)**

1. differs between YOY or 1-year-old calf versus 2-year-old calf

**from(2 3,4)**

1. equal for all young

**i**

**Step 3: Ageing**

No parameters were estimated in this matrix as changes in age class for calves was forced to 1 (*) between *t* and *t+1*. Gemaco information: **i**

**Step 4: Breeding**

1. all states different

**from**

1. differs between non-breeding females versus females that lost a YOY or a calf versus females that raised a calf to the age of 3

**from(1,5 6 7,8)**

1. differs between non-breeding females versus females that lost a YOY or a 1-year-old calf versus females that lost a 2-year-old calf or raised a calf to the age of 3

**from(1, 5 6, 7 8)**

1. differs between non-breeding females and females that lost a 2-year-old calf or raised a calf to the age of 3 versus females that lost a YOY or a 1-year-old calf

**from(1 5 6, 7 8)**

1. differs between non-breeding females versus females that had offspring (dead or raised)

**from(1,5:8)**

1. breeding probability equal for all states

**i**

**Detection**

We included a temporal variation (t) in an additive way for the detection probability, as sampling was not equal between years.

1. all states different

**firste+nexte.[from+t]**

1. differs between non-breeding females versus females with a YOY versus females with a calf (age 1 to 3)

**firste+nexte.[from(1,2,3 4 5)+t]**

1. differs between non-breeding females versus females with a YOY or a 1-year-old calf versus females with a 2- or 3-year-old calf

**firste+nexte.[from(1,2 3,4 5)+t]**

1. differs between non-breeding females or females with a 2- to 3-year-old calf versus females with a YOY or 1-year-old calf

**firste+nexte.[from(1 4 5,2 3)+t]**

1. differs between non-breeding females versus females with offspring (YOY or calf)

**firste+nexte.[from(1,2:5)+t]**

1. detection probability equal for all states

**firste+nexte.[i+t]**

**Observation**

1. all states different

**from**

1. differs between a breeding female with a YOY versus a breeding female with a calf (age 1 to 3)

**from(3, 4 5 6)**

1. differs between a breeding female with a YOY or a 1-year-old calf versus a breeding female with an older calf

**from(3 4,5 6)**

1. equal for every breeding female

**i**

**APPENDIX S4**: For each year of the study (from 2004 to 2016, in the period between July and November), details on event occurrence (event 1 = female sighted alone; event 2 = female sighted with a young-of-the-year; event 3 = female sighted with a calf between the ages of 1 and 3), the number of sighted females (a total of 106 females were sighted over the full study period), and the number of identification photos analyzed.

| Year | Occurrence event 1 | Occurrence event 2 | Occurrence event 3 | Sighted females | Number of photos |
| --- | --- | --- | --- | --- | --- |
| 2004 | 10 | 0 | 1 | 11 | 74 |
| 2005 | 11 | 1 | 7 | 19 | 423 |
| 2006 | 13 | 0 | 7 | 20 | 275 |
| 2007 | 35 | 19 | 6 | 60 | 1793 |
| 2008 | 24 | 7 | 19 | 50 | 1057 |
| 2009 | 49 | 7 | 22 | 78 | 2229 |
| 2010 | 48 | 18 | 21 | 87 | 3179 |
| 2011 | 35 | 15 | 28 | 78 | 1179 |
| 2012 | 36 | 8 | 17 | 61 | 666 |
| 2013 | 32 | 15 | 23 | 70 | 1239 |
| 2014 | 29 | 11 | 11 | 51 | 839 |
| 2015 | 39 | 9 | 8 | 56 | 283 |
| 2016 | 21 | 4 | 8 | 33 | 111 |
| Total | 382 | 114 | 178 | 674 | 13347 |
